# The Novel Coronavirus COVID-19 Outbreak: Global Implications for Antimicrobial Resistance

**DOI:** 10.3389/fmicb.2020.01020

**Published:** 2020-05-13

**Authors:** Aimee K. Murray

**Affiliations:** European Centre for Environment and Human Health, University of Exeter Medical School, Environment & Sustainability Institute, Penryn, Cornwall, United Kingdom

**Keywords:** coronavirus, COVID-19, antibiotic resistance, antimicrobial resistance, antibiotics

## Introduction

We are in the midst of the novel coronavirus (COVID-19) pandemic, the most significant global health event since Spanish influenza in the early 20th century. Increasingly draconian measures are being implemented worldwide to try to slow the spread of the virus. Antimicrobial resistance (AMR) has been cited as the most significant threat to the global health and global economy in recent years, but is now likely to be eclipsed by COVID-19 for some time. However, the emergence of COVID-19 also presents some important consequences for the development of AMR. This piece will highlight how managing the COVID-19 crisis could impact AMR in the clinic, beyond the clinic in the community, in the environment and in relation to public awareness. When civilization emerges from the other side of this global health emergency, efforts should be made to understand these potential effects on AMR, the other significant, and constant global health issue of our time.

### In the Clinic

Healthcare systems around the world are under increasingly immense pressure. This is leading to several changes in practice that may have impacts on, or relevance to, AMR.

For example, the UK government have published several documents relating to COVID-19 management in clinical settings. In the guidance for primary care, it is recommended that any room that has been used for a patient with a suspected SARS-CoV-2 (the causative agent of COVID-19) infection should remain closed and ventilation switched off until full sterilization has taken place (HM Government, 2020a). With regards to infection prevention and control procedure, additional measures are recommended regarding transmission prevention. These include precautions around direct contact with potentially contaminated surfaces, droplets and aerosols (HM Government, 2020b). These may not become routine management options within clinical settings following the COVID-19 pandemic, however, many of these practices may also reduce dissemination of AMR bacteria at a local and global scale. In particular, extra vigilance around hygiene and additional sterilization procedures may reduce the spread of AMR bacteria. It would be interesting to gather data on the prevalence of AMR infections before and after the outbreak to determine if this is the case. Comparison of whole genome sequences of clinical pathogens before, during and after the pandemic is one potential technique that could elucidate changes in carriage of AMR mechanisms circulating in clinical settings. Databases such as BacWGSTdb (Ruan and Feng, 2016) could also be used to track outbreaks of key AMR pathogens to the species, clonal complex or isolate level.

With regards to COVID-19 patients contracting secondary bacterial infections, there are very few data so far. However, 1 to 10% of patients have been reported to contract secondary bacterial infections in two separate studies (Lai et al., 2020). This in comparison to infection with pandemic H1N1, where around 12–19% of hospitalized patients with pneumonia developed secondary bacterial infections (Kim, 2020). Given current data it is not possible to predict whether the cases of secondary bacterial infection following development of COVID-19 will increase or decrease overtime. Clinical microbiologists, as well as radiologists, will be key for making these distinctions (Kim, 2020). However, despite the relatively low confirmation of secondary bacterial infections, there have been comparatively more reports of antibiotic usage when treating COVID-19 patients (Lai et al., 2020), including up to 45% of patients receiving antibiotic treatment (Xu et al., 2020). This is even though the World Health Organization recommended against the use of antibiotics during COVID-19 treatment (Cascella et al., 2020). It has also been suggested that certain antibiotics, such as tecioplanin (a glycopeptide antibiotic) could be used as an antiviral after exhibiting activity against coronaviruses (amongst others) previously (Baron et al., 2020). However, great caution should be used given that inappropriate use or overuse of antibiotics is known to be a significant driver of the emergence of AMR. This is why significant focus on AMR revolves around reducing inappropriate or overuse of antibiotics (NICE, 2018). Countries which have made progress in this area may face less AMR secondary bacterial infections than countries that have experienced limited success in reducing antibiotic consumption. Again, it would be interesting to analyse this data, when available. The second reason use of antibiotics should be considered very carefully is that it may lead the public to assume that all antibiotics are suitable for treatment of viral infections (see “Public Awareness,” below).

### Beyond the Clinic

Outside the clinic, countries are employing measures aimed at reducing transmission of COVID-19 that range from social distancing, to full-on lock down and closing borders. One piece of advice to the public that has remained constant from the beginning, however, is for the public to regularly wash their hands with soap and water (or to use hand sanitiser, when these are unavailable).

Use of antimicrobial soaps and disinfectant cleaners by members of the community and in the hospital will have increased hugely over the last few months. Higher usage is likely to continue, and may even remain high following the outbreak due to changes in infection and control policy or individual habits. As discussed above, these increased/improved hygiene practices may reduce the spread of AMR, which is a very positive outcome. However, there is also a potential negative impact that could arise from increased use of such products, as many of them contain biocides. Biocides are antimicrobials found in surface disinfectants and household cleaners (Buffet-Bataillon et al., 2012) that may also lead to the emergence of AMR (Levy, 2002; Maillard, 2005; Pal et al., 2015; Webber et al., 2015). Due to the COVID-19 pandemic, higher concentrations of biocides are likely to be detected in wastewater treatment plants and receiving waters. This may increase levels of AMR in the environment, posing a human health risk for individuals exposed to these environments. The final concentration of biocide in the wastewater treatment plant and its receiving environments is key. If very high, it is likely most bacteria will be completely inhibited. This could cause significant impacts on key ecosystem services performed by bacteria but prevent the selection for or development of AMR. Conversely, if concentrations increase but remain below the minimum inhibitory concentration for the majority of bacteria present, this increase in selective pressure could provide an opportunity for the evolution of AMR (McBain et al., 2002). The phenomenon of sub-inhibitory selection is comparatively well-studied for antibiotics, with significantly fewer experimental studies on biocides. Increased antibiotic consumption to treat or prevent secondary bacterial infections in COVID-19 patients, or as a potential therapy for COVID-19, will also result in increased concentrations of antibiotics in the wastewater system and receiving environments. Again, this increased selective pressure may result in selection for AMR. However, unlike with biocides, it is highly doubtful that completely inhibitory concentrations of antibiotics could be reached, due to metabolism by the patient and a greater dilution factor. Furthermore, it has been shown previously that low concentrations of antibiotics can select for AMR just as much as high, clinically relevant concentrations (Murray et al., 2018). These increased concentrations of biocides and antibiotics in wastewater as a result of the COVID-19 pandemic and their impacts would form an interesting area of research.

Significant reductions in travel (in addition to resulting in a much-needed reduction in carbon dioxide emissions) will also have impacts for the spread of AMR. Movement of key AMR genes between countries in undeniable. For example, one of the key genes conferring resistance to last resort carbapenem antibiotics (*NDM-1*) was first isolated in India (Liang et al., 2011), and has since been detected worldwide (Nordmann et al., 2011). Similarly, emergence of the *mcr1* gene that confers resistance to another last resort antibiotic, colistin, was first detected in China (Liu et al., 2016) but has since been found worldwide (Castanheira et al., 2016). Transferable tigecycline resistance gene *tet*(X4) was also detected in China for the first time last year (Bai et al., 2019). The *CTX-M* genes originated in environmental bacteria (Humeniuk et al., 2002; Olson et al., 2005; Cantón et al., 2012) but have since been labeled a “pandemic” (Canton and Coque, 2006). Whilst a viral pandemic has the more immediate outcome of infection, often with symptoms, transmission of AMR may result in infection, or colonization and shedding. For example, it has been shown that following travel to countries with high rates of AMR, travelers can become colonized by new AMR genes or bacteria. Following travel to China, India or northern Africa, colonization of Swedish travelers with extended-spectrum beta-lactamase producing Enterobacteriaceae increased from 2.4 to 68%, and this took weeks to months (and up to 1 year) to return to a pre-travel level (ÖstholmBalkhed et al., 2018). Reduction of travel on such a massive scale should have also slowed the spread of AMR.

### Public Awareness

There is no denying the understandably extensive media coverage of the COVID-19 pandemic. In particular, how the outbreak has crossed international borders so rapidly to become the current crisis facing all countries. AMR has been reportedly described as a problem that “knows no borders.” According the WHO, the definition of a pandemic is human-to-human spread of microorganisms and community-level outbreaks in three countries, one of which must be within a different WHO region (WHO, 2009). Arguably, AMR can also be considered as a pandemic, although a more insidious one that has fewer immediate effects on everyday life but potentially more far reaching negative impacts. According to the European Center for Disease Control and Prevention, at the time of writing, 190, 236 lives have sadly been lost to COVID-19 globally over the past 4 months (ECDC, 2020). AMR currently kills an estimated 700, 000 people each year (IACG, 2019). For a crude comparison, assuming both figures are accurate estimates and COVID-19 death rates remain constant for the remainder of the year, AMR will result in 130,000 more deaths this year alone. In addition, AMR deaths are predicted to increase to 10 million deaths per year by 2050 (O'Neill, 2014), whereas it is hoped COVID-19 can be managed in a much shorter time frame.

In future, COVID-19 may be a useful comparison for describing the spread of AMR and highlighting how difficult it is to control, once it has emerged. According to a study performed by the WHO, a very common misconception amongst the public is that antibiotics can be used for viral infections (i.e., the common cold) (WHO, 2015). Media coverage of the COVID-19 outbreak has highlighted there is no “cure” for infection, often stating antibiotics are ineffective and antiviral treatments are being trialed in certain countries. Using terms like “antiviral” may also help with understanding there are different medications for different types of infection. Furthermore, people who are self-isolating due to suspected or confirmed infection with COVID-19 may have previously asked for antibiotics. If they have adhered to the self-isolation protocol, they would not have been able to visit their family doctor to request such a prescription. It is possible that the public may now have greater awareness of suitable use of antibiotics, which should be capitalized on once the outbreak has been controlled. A long-term, potential benefit could be reduced antibiotic use that should be considered when discussing potential antibiotic therapies for COVID-19. Repeating studies that examine public understanding of appropriate antibiotic use, such as the one above, would be useful to see if the outbreak has caused a shift in public awareness of AMR.

### Conclusions

Potential implications, both good and bad, of some of the current management practices and practicalities of managing the novel coronavirus outbreak in relation to AMR have been discussed. This is by no means a comprehensive list and without doubt, further impacts will become apparent as the situation rapidly progresses. This pandemic will be considered a significant event in human history. Both emerging infectious diseases and AMR are included in the UK government's National Risk Registry of Civil Emergencies (HM Government, 2017). The global issue of AMR will persist beyond the COVID-19 outbreak, and understanding some of the impacts the management strategies employed globally had, or will have, on AMR in the clinic, the environment and regarding public awareness should be investigated, when the time is right. In the mean time, everyone should wash their hands.

## Author Contributions

The author confirms being the sole contributor of this work and has approved it for publication.

## Conflict of Interest

The author declares that the research was conducted in the absence of any commercial or financial relationships that could be construed as a potential conflict of interest.
